# Influence of the Host and Parasite Strain on the Immune Response During *Toxoplasma* Infection

**DOI:** 10.3389/fcimb.2020.580425

**Published:** 2020-10-15

**Authors:** Debanjan Mukhopadhyay, David Arranz-Solís, Jeroen P. J. Saeij

**Affiliations:** Department of Pathology, Microbiology & Immunology, School of Veterinary Medicine, University of California, Davis, Davis, CA, United States

**Keywords:** *Toxoplasma*, immune response, strain, virulence factor, ileitis, encephalitis

## Abstract

*Toxoplasma gondii* is an exceptionally successful parasite that infects a very broad host range, including humans, across the globe. The outcome of infection differs remarkably between hosts, ranging from acute death to sterile infection. These differential disease patterns are strongly influenced by both host- and parasite-specific genetic factors. In this review, we discuss how the clinical outcome of toxoplasmosis varies between hosts and the role of different immune genes and parasite virulence factors, with a special emphasis on *Toxoplasma-*induced ileitis and encephalitis.

## Introduction

*Toxoplasma gondii* is an obligate intracellular parasite and the causative agent of toxoplasmosis, a worldwide zoonotic disease that can affect virtually all mammals and birds, including approximately one third of humans (Montoya and Liesenfeld, 2004; Pappas et al., 2009). *Toxoplasma* has a complex life cycle with sexual multiplication taking place exclusively in the intestine of the definitive host (members of the felidae family) and asexual multiplication in intermediate hosts (warm-blooded animals). Infection of intermediate hosts occurs by ingestion of food or water contaminated with oocysts, which are secreted with feline feces, or meat containing tissue cysts. Upon ingestion, sporozoites within oocysts or bradyzoites within tissue cysts are released in the small intestine and invade intestinal epithelial cells. Here they transform into tachyzoites, the fast-replicating stage responsible for symptoms during the acute phase of the disease, which can vary in severity depending on the host and parasite strain. After invasion of the host cell, *Toxoplasma* establishes its niche inside the host cytosol by forming a parasitophorous vacuole (PV), inside which it replicates (Blader et al., 2015). *Toxoplasma* modulates host pathways through the secretion and delivery of GRA and ROP effectors from its dense granule and rhoptry organelles, respectively, to the host cell cytoplasm. Although some *Toxoplasma* secreted GRAs and ROPs help to evade host immunity (Hunter and Sibley, 2012; Butcher et al., 2014; Hakimi and Bougdour, 2015; Hakimi et al., 2017; Boothroyd and Hakimi, 2020), others activate it (Melo et al., 2011; Hunter and Sibley, 2012; Hakimi et al., 2017; Boothroyd and Hakimi, 2020). This dynamic balance is required by the parasite to convert to bradyzoites and form tissue cysts, which are orally infectious to other intermediate hosts, thus favoring further transmission. Hence, a complete evasion of host immunity leading to uncontrolled parasite proliferation would be fatal for the host, while a too strong host immune response would be fatal for the parasite, either of which would limit the parasite's transmission.

Most hosts with competent immune responses develop only mild flu-like symptoms or remain asymptomatic, as they are capable of controlling parasite multiplication. By contrast, susceptible host species or individuals with immunosuppressed conditions develop clinical toxoplasmosis that may manifest as encephalitis, myocarditis or pneumonia, among others. In addition, infection during gestation can result in abortion, neonatal mortality or congenital infection (Dubey, 2016).

### Susceptibility to the Disease Is Determined by Both Parasite Strain and Host Species

The immune response against *Toxoplasma* varies depending on the genetic background and immune status of the host, as well as on the parasite strain. In Europe and North America, the population of *Toxoplasma* is dominated by four major clonal types, I, II, III, and XII, although the majority of human and livestock infections in these regions are caused by type II strains (Howe and Sibley, 1995; Ajzenberg et al., 2002; Sibley and Ajioka, 2008; Lorenzi et al., 2016). However, in South America *Toxoplasma* shows an extreme genetic diversity with more than 150 strains (Sibley and Ajioka, 2008; Su et al., 2012). *Toxoplasma* virulence is defined by the number of tachyzoites that are needed to kill 50% of the mice upon inoculation (LD_50_). In the house mouse (*Mus musculus -M.m.- domesticus*) type I strains are very virulent (LD_100_ of 1 parasite), whereas type II and III are less virulent strains (LD_50_ ~10^3^ and ~10^5^ parasites, respectively) (Sibley and Boothroyd, 1992; Saeij et al., 2006). However, little is known about the correlation between *Toxoplasma* strain virulence in mice compared to other animal species and humans (Saeij et al., 2005). For instance, while type I strains are highly virulent in most inbred murine hosts, they fail to produce observable disease in other animals such as rats or cows.

Generally, there seems to be a trend for *Toxoplasma* strains that are abundant in specific regions to not be virulent for the native mouse subspecies from those regions. For example, type I strains are more prevalent in North and Southeast Asia (Shwab et al., 2014), where *M. m. castaneus* and *M.m. musculus* are also more abundant. Type I strains do not kill these mice but are extremely virulent to *M.m. domesticus*, which is the dominant subspecies in Europe and North-America. By contrast, type II and III strains, which predominate in Europe and North-America, generally do not kill *M.m. domesticus*. South-American *Toxoplasma* strains are mostly virulent in mice, which are not indigenous to the continent, and these strains might have adapted to South-American rodents (Lilue et al., 2013; Hassan et al., 2019; Murillo-León et al., 2019). The difference in susceptibility against *Toxoplasma* in these different mouse (sub)species has been mapped to chromosome 11, which contains the immunity related GTPases (IRGs), a family of proteins that are induced by interferon gamma (IFNγ) and which coat and subsequently vesiculate the parasitophorous vacuole membrane (PVM), leading to the death of the parasite (Lilue et al., 2013; Hassan et al., 2019; Murillo-León et al., 2019). These IRG proteins are highly polymorphic and their function is blocked by the *Toxoplasma* effectors ROP5 and ROP18 (Fentress et al., 2010; Fleckenstein et al., 2012; Niedelman et al., 2012; Lilue et al., 2013). Thus, an evolutionary arms race of reciprocal selection pressure might drive the polymorphisms of IRGs in mice vs. ROP5 and ROP18 in *Toxoplasma*. Several inbred and outbred murine models have also been shown to have different degrees of susceptibility, which has been mostly investigated after infection with type II strains (Johnson, 1984; Shirahata et al., 1986; McLeod et al., 1989a,b, 1993; Schade and Fischer, 2001; Lee and Kasper, 2004). For instance, A/J inbred mice are relatively resistant regardless of the route of infection, showing very low cyst numbers in the brain and minimal inflammation and necrosis in the small intestine upon oral infection (McLeod et al., 1989a,b; Jensen et al., 2013). This is possibly due to the increased parasite killing ability of A/J derived macrophages compared to C57BL/6 (Hassan et al., 2015), although the differences in cyst numbers are also influenced by the Histocompatibility system 2 (H-2) locus (H-2a for A/J and H-2b for C57BL/6) (McLeod et al., 1989b). Other wild rodents, such as voles, are common in certain areas and are more resistant than mice (Sedlák et al., 2001). Similarly, guinea pigs show only a moderate susceptibility to type I and type II *Toxoplasma* infections (Flori et al., 2002).

Evidence from other animals also suggests a species-dependent susceptibility to toxoplasmosis. For instance, Australian marsupials, especially wallabies, and new world monkeys are particularly susceptible to toxoplasmosis, which often proves fatal (Innes, 1997). These species have either largely evolved in the absence of felines or their habitat is high in the canopies of trees, thus preventing them from being exposed to oocysts on the ground, which may explain their high vulnerability. Other animals are more resistant to *Toxoplasma* infection, with the infection usually being inapparent or producing only transient mild symptoms during the acute phase, although the host remains chronically infected for its lifetime. This group includes sheep, pigs, and humans, among others. Bovines are more resistant than other farm animals (Innes, 1997; Stelzer et al., 2019). In addition, although most types of rats are resistant to the acute phase of infection by the major *Toxoplasma* clonal types, rats often develop brain cysts (Kempf et al., 1999; Sergent et al., 2005). However, Lewis rats have sterile immunity against most *Toxoplasma* strains, which is a dominant trait mapped to the *Toxo*1 locus (Cavaillès et al., 2006, 2014). This locus contains a nucleotide-binding oligomerization domain, leucine-rich repeat protein 1 (*NLRP1*), which encodes for the NLRP1 inflammasome sensor that causes rapid cell death and disruption of the replicative niche of the parasite upon activation (Cirelli et al., 2014). One exception is the South American strain GUY008-ABE, which can establish a chronic infection in Lewis rats and is fatal to F334 rats (Loeuillet et al., 2019). Thus, although it is in the parasite's best interest to cause a lifelong chronic infection, it is clear that not every parasite strain can produce an optimal infection in every animal.

Arguably, one of the most critical factors influencing susceptibility to *Toxoplasma* is the host immune system and its interplay with the parasite. For example, it is possible that in highly susceptible species the induction or function of critical components of protective immunity is less efficient than in more resistant species. As natural infection in immunocompetent humans is mostly asymptomatic, studies addressing the human immune response in primary toxoplasmosis are challenging. Furthermore, the immune response in humans also varies with the clinical presentation, ranging from toxoplasmic ileitis to toxoplasmic encephalitis (TE), ocular toxoplasmosis (OT) or congenital toxoplasmosis (Montoya and Liesenfeld, 2004). To study different clinical manifestations of toxoplasmosis in the laboratory with relative ease, various rodent models have been developed and provided much of our understanding of toxoplasmosis immunobiology (Munoz et al., 2011; Dupont et al., 2012). In this review we will discuss the immune response against *Toxoplasma*, focusing on parasite and host factors that influence *Toxoplasma-*induced ileitis and encephalitis.

## The Immune Response During Toxoplasmosis

The immune response against invading microorganisms starts with the sensing of pathogens via microbe associated molecular patterns (PAMPs/MAMPs) by receptors known as pattern recognition receptors (PRRs). The toll-like receptors (TLRs) and nucleotide-binding oligomerization domain-like receptors (NLRs) are the most extensively studied (Akira et al., 2006; O'Neill and Bowie, 2007; Mogensen, 2009). Upon engagement of TLRs or NLRs with MAMPs, signaling pathways are triggered. Downstream signal transduction pathways via phosphorylation, ubiquitination, or protein-protein interactions, activate host cell transcription factors which in turn upregulate the expression of genes involved in inflammation and antimicrobial host defenses (Akira et al., 2006; O'Neill and Bowie, 2007; Mogensen, 2009). Three major signaling pathways are responsible for mediating TLR or NLR-induced immune responses: (i) nuclear factor κB (NF-κB), (ii) mitogen-activated protein kinases (MAPKs), and (iii) IFN regulatory factors (IRFs). These three pathways play central roles in the induction of interleukin (IL)12, IL1β, tumor necrosis factor α (TNFα) and interferons (IFN) type I and type II (Akira et al., 2006; O'Neill and Bowie, 2007; Mogensen, 2009). The activation of these pathways leads to the recruitment of immune cells to the site of infection via the secretion of chemokines, and these recruited cells can kill *Toxoplasma* via the upregulation of effector molecules. There are important differences in how the innate immune system of different host species detect *Toxoplasma* and, once it is detected, how it is eliminated via IFNγ-induced anti-parasitic activities.

### Host Differences in the Innate Sensing of *Toxoplasma*

#### Role of Toll Like Receptors (TLRs)

**Figure 1:**
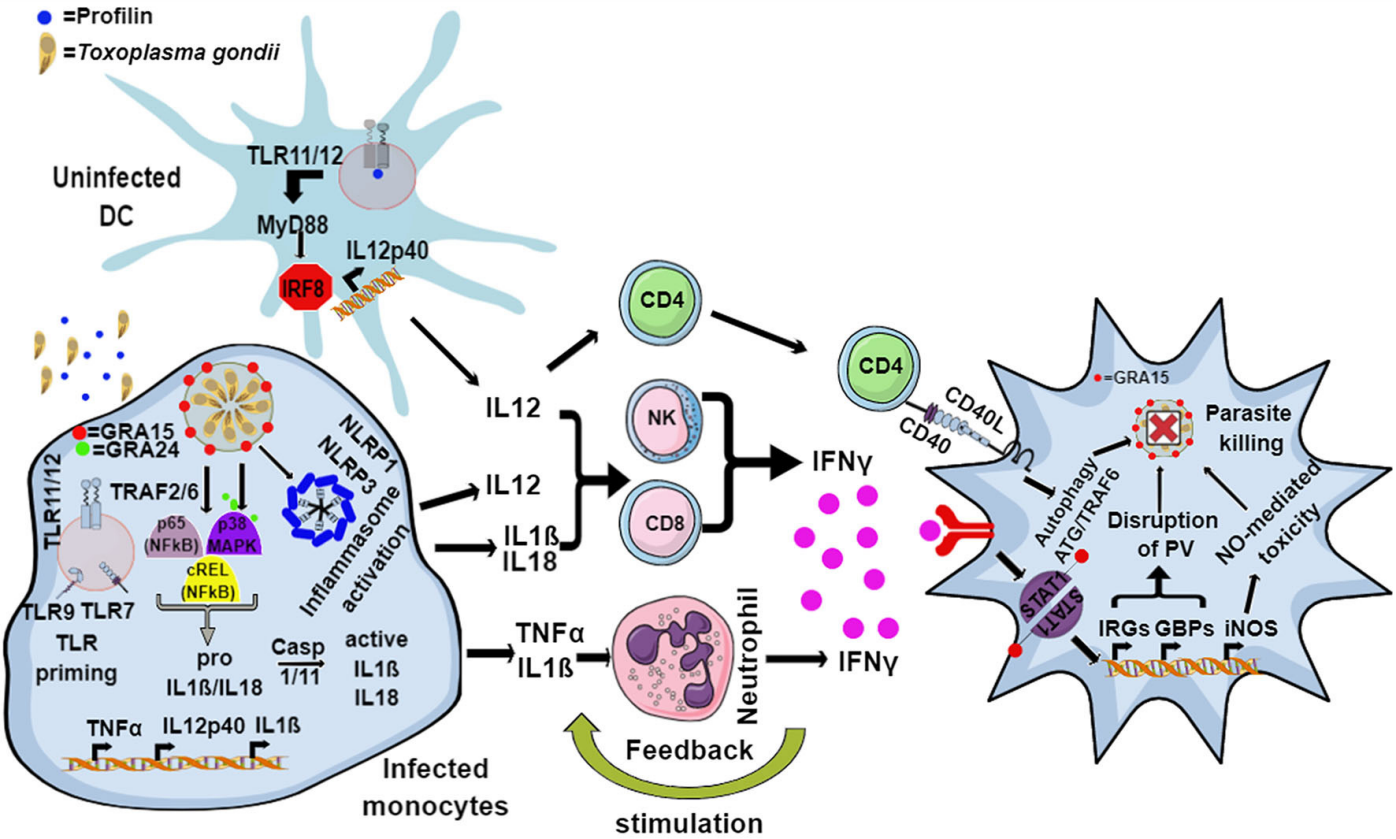
Model for the murine immune response to *Toxoplasma*. The endosomal TLR11 and TLR12 heterodimeric complex in uninfected DCs recognizes *Toxoplasma* profilin, resulting in MyD88 and IRF8-mediated production of IL12. Infected monocytes and macrophages produce IL1β and IL18 through the activation of the NLRP1 and NLRP3 inflammasome, while secretion of IL12 and TNFα is induced by GRA15- and GRA24-mediated activation of p65-NFκB, cREL-NFκB, and p38 MAPK pathways. Additionally, priming through nucleic acid sensing TLRs also leads to induction of IL1β. IL12, along with IL18 and IL1β, activates NK, CD4, and CD8 T cells to produce host-protective IFNγ. IL1β and TNFα act together to activate incoming neutrophils to secrete IFNγ, in turn inducing the expression of a battery of effector molecules including the immunity related GTPases (IRGs), guanylate binding proteins (GBPs), reactive oxygen species (ROS), nitric oxide (NO), and autophagy pathways inside the infected cells that eliminate the parasite.

In murine hosts, *Toxoplasma* is primarily detected by endosomal TLR11 and TLR12, which bind to the *bona-fide Toxoplasma* MAMP actin-binding protein, profilin in uninfected dendritic cells (DCs) (Yarovinsky, 2005; Andrade et al., 2013; Raetz et al., 2013). This interaction signals via myeloid differentiation factor 88 (MyD88) and activates the IRF8 transcription factor, resulting in the secretion of large amounts of interleukin 12 (IL12) (Yarovinsky, 2005, 2014; Andrade et al., 2013; Raetz et al., 2013) (Figure 1). However, the TLR11/12-profilin interaction is largely restricted to the CD8α subset of DCs, and by what means other immune cells, like macrophages, sense the pathogen remains elusive. In addition, the endosomal RNA and DNA sensing TLR7 and TLR9 are also important for the secretion of IL12, as 3d mice (which lack the chaperone UNC93B1 that is important for the correct localization of TLR 3/7/9/11/12) and *Tlr3/7/9/11*^−/−^ quadruple knockout mice (Melo et al., 2010; Andrade et al., 2013) are very susceptible to infection (Figure 1).

Many other hosts, including humans, lack functional TLR11/12 (Gazzinelli et al., 2014) (Table 1) suggesting that these hosts have other mechanisms to detect *Toxoplasma*. Most hosts express TLR7, TLR9 and other nucleic acid sensors such as “absent in melanoma protein 2” (AIM2), cyclic GMP-AMP synthase (cGAS) or retinoic acid-inducible gene I (RIG-I). It is likely that only when the PVM is destroyed, parasite-derived nucleic acids are released and detected by cytoplasmic nucleic acid sensors. Indeed, human peripheral blood mononuclear cells (PBMCs) do not secrete cytokines upon treatment with *Toxoplasma* derived RNA or DNA unless pre-stimulated with IFNγ (Andrade et al., 2013). Cell surface TLR2 can sense the glycosylphosphatidylinositol (GPI) anchors on the parasite surface; however it does not seem to be essential for cytokine production by DC (Scanga et al., 2002; Debierre-Grockiego et al., 2007). Nevertheless, TLR2 was shown to be essential for the survival of infected mice, especially at high doses of infection, a scenario also observed in *Mycobacterium tuberculosis* (Reiling et al., 2002; Mun et al., 2003). Therefore, further studies are needed to underpin the role of TLR2 in *Toxoplasma* infection. Human and bovine TLR5 are closely related to murine TLR11; however, the response triggered by engagement of *Toxoplasma-*derived profilin with bovine and human TLR5 is controversial. While one study showed that human TLR5 binds to profilin and is able to stimulate the cells to secrete pro-inflammatory cytokines (Salazar Gonzalez et al., 2014), another study showed that human and porcine TLR5 failed to trigger cytokine secretion from cells upon stimulation with profilin (Tombácz et al., 2018). The discrepancy might be due to the purity of the profilin used. Although information from humans is scarce, a recent study showed that human monocytes can sense *Toxoplasma* in an indirect way (Safronova et al., 2018). Lysis of the infected cells releases the alarmin S100A11 through the inflammatory caspase 1 dependent pathway, which is sensed by monocytes through the receptor of advanced glycation end product (RAGE). This results in the production of the monocyte chemoattractant protein 1(MCP1), also called C-C motif chemokine ligand 2 or CCL2, which in turn recruits more monocytes, DCs and T cells to the site of infection (Safronova et al., 2018).

**Table 1:** Immune genes that can induce toxoplasmacidal mechanisms and their functional status in different hosts.

**Species**	**NLRP1**	**NLRP3**	**P2X_**7**_R**	**TLR9**	**TLR11**	**GBP1**	**GBP2**	**GBP5**	**IRG**
Human	✓^$^	✓	✓	✓	×	✓	✓	✓	×^*^
Mice	✓	✓	✓	✓	✓	✓	✓	✓	✓
Rat	✓	✓	✓	✓	✓	✓	✓	✓	✓
Bovine	✓	✓	✓	✓	×	Truncated	✓	✓	×
Ovine	✓	✓	✓	✓	×	×	×	✓	×
Porcine	✓	✓	✓	✓	✓	✓	✓	×	×
Equine	✓	✓	✓	✓	✓	✓	✓	✓	×
Canine	✓	✓	✓	✓	×	✓	×	✓	✓
Feline	×	✓	✓	✓	×	×	×	×	×
Chicken	Truncated	✓	×	×	×	✓	×	×	×

$ *Only human and canine NLRP1 have an N-terminal pyrin domain (PYD) while all other variants have a C-terminal caspase activating and recruitment domain (CARD)*.

* *Humans have only one regulatory IRGs but no effector IRGs (Gazzinelli et al., 2014)*.

#### Role of Inflammasomes in Sensing Parasites

In mammals or mice lacking TLR11, activation of the immune system upon *Toxoplasma* infection is dependent on cytoplasmic sensors such as NLRP1/3, the adaptor protein apoptosis associated speck-like protein containing a CARD (ASC) and the caspases 1 and 4, all of which lead to the secretion of IL1β and IL18, a process known as inflammasome activation (Witola et al., 2011; Gov et al., 2013, 2017; Mukhopadhyay et al., 2020a). In mice lacking TLR11, and therefore DC-derived IL12, a large amount of IFNγ is secreted by neutrophils and CD8 T cells via pro-inflammatory monocyte and macrophage dependent inflammasome driven secretion of IL1β and IL18 (Sturge et al., 2013; López-Yglesias et al., 2019) (Figure 1). Indeed, mice deficient in components of the inflammasome sensors NLRP1, NLRP3, ASC, caspase 1 and caspase 11, or downstream cytokine IL18 or its receptor (IL18R) all showed increased susceptibility to *Toxoplasma* infection (Gorfu et al., 2014). In line with this, in human PBMCs, both IL18 and IL1β secreted through the activation of the NLRP3 inflammasome pathway are responsible for the secretion of IFNγ and IL12 (Mukhopadhyay et al., 2020a). Furthermore, apart from murine models, inflammasome activation is also important to restrict parasite growth in human and rat macrophages (Sergent et al., 2005; Witola et al., 2011; Cavaillès et al., 2014; Cirelli et al., 2014; Gov et al., 2017; Fisch et al., 2019a). Polymorphisms in NLRP1 determine susceptibility to toxoplasmosis in rats and humans but not in mice (Witola et al., 2011; Ewald et al., 2014). However, other hosts such as felines and chickens do not possess NLRP1 and are not susceptible to the disease (Table 1). This highlights the fact that other PRRs may also elicit an effective immune response against *Toxoplasma*. NLRP3 and the purinergic receptor P2X_7_R, which also leads to inflammasome activation, are widely expressed in rodents, humans and other animals (Table 1). These receptors play an important role in the control of toxoplasmosis, as their deficiency or polymorphisms produce susceptibility to the disease (Corrêa et al., 2010; Lees et al., 2010; Miller et al., 2011; Gorfu et al., 2014) (Figure 1).

### Role of Cytokines and Chemokines in Recruitment and Activation of Immune Cells

Central to the host defense against toxoplasmosis is the generation of IL12 by DCs and macrophages, as it primes NK and T cells (CD4 and CD8) to secrete IFNγ. Although DC-derived IL12 is important for secretion of IFNγ *in vivo*, it alone is not sufficient to protect the host, as *Myd88*^−/−^ mice that were administered recombinant IL12 post-infection did not survive infection (Hou et al., 2011). The pro-inflammatory cytokines IL18 and IL1β secreted by monocytes and macrophages upon inflammasome activation also enhance IFNγ production and, together with IL12, can thereby contribute to host resistance against *Toxoplasma* (Hunter et al., 1995; Cai et al., 2000; Gov et al., 2013, 2017; Mukhopadhyay et al., 2020a). Furthermore, while mice lacking either MyD88 or IL12p40 succumb rapidly to infection, mice that lack Myd88 specifically in DCs or macrophage lineages do not, indicating that there are other factors and cytokines that regulate IL12 and IFNγ secretion (Scanga et al., 2002; Hou et al., 2011). As a matter of fact, neutrophils recruited to the site of infection secrete IL12 and IFNγ, while monocytes contribute by secreting IL1β and TNFα, which in turn induce the secretion of IFNγ by neutrophils, NK cells and T cells in a MyD88-dependent way (LaRosa et al., 2008; Sturge et al., 2013; Sturge and Yarovinsky, 2014). Therefore, recruitment of immune cells to the site of infection via chemokine secretion is considered an essential step in the control of the parasite. For instance, NK cells are recruited to the site of infection through the CC chemokine receptor 5 (CCR5) and are among the first cells to secrete IFNγ, while neutrophils and monocytes are recruited through CCR1 and CCR2, respectively (Khan et al., 2001, 2006; Robben et al., 2005). The importance of immune cell recruitment was revealed by the acute susceptibility of mice lacking either CCR5, CCR1, or CCR2 (Khan et al., 2001, 2006; Robben et al., 2005; Dunay et al., 2008). Cooperation between DCs and NK cells further stimulates IL12 and IFNγ production by each cell type (Khan et al., 2006; Guan et al., 2007). The importance of CD8α DCs and NK cells was revealed from experiments conducted in mice lacking CCR5, IRF8, or basic leucine zipper transcription factor ATF-like 3 (BATF3), a transcription factor required for the development of CD8α DCs, as all of them succumb quickly to the infection (Scharton-Kersten et al., 1997; Khan et al., 2006; Mashayekhi et al., 2011).

### Elimination of Parasites Within the Host

IFNγ, alone and together with TNFα, induces the secretion of effector molecules in both hematopoietic and non-hematopoietic cells, eliciting a direct anti-parasitic activity that will be discussed in the next section. Although IFNγ is indispensable for mouse immunity to *Toxoplasma* (Suzuki et al., 1988; Scharton-Kersten et al., 1996; Yap and Sher, 1999), humans with IFNγ-receptor deficiency do not show an increased susceptibility. This could be possibly due to TNFα and CD40-CD40L-mediated (Janssen et al., 2002; Andrade et al., 2003) induction of host protective autophagy in infected macrophages and non-hematopoietic cells, leading to parasite elimination (Andrade et al., 2003, 2006; Subauste and Wessendarp, 2006; Van Grol et al., 2013) (Figure 1). Neutrophils, the most abundant cells at the site of infection, can form neutrophil extracellular traps, or NETs. These consist of extracellular masses of nuclear DNA fibers associated with histones and enzymes of cytoplasmic granules and anti-microbial peptides, such as myeloperoxidase (MPO) or lactoperoxidase, that are able to kill various microorganisms (Brinkmann et al., 2004; Díaz-Godínez and Carrero, 2019). In the case of *Toxoplasma*, NETs cause the immobilization of entrapped parasites, leading to their death (Abdallah et al., 2012; Yildiz et al., 2017). This mechanism is conserved in mice, humans, farm animals and marine mammals (Abdallah et al., 2012; Yildiz et al., 2017; Imlau et al., 2020).

After the initial sensing, immune activation and clearance of parasites within macrophages and other antigen presenting cells (APCs), the CD8 T cell response is initiated when they encounter epitopes presented by major histocompatibility complex (MHC) I on APCs. The degree of CD8 T cell activation depends on the effective killing of intracellular parasites within the APCs, after which the immunodominant epitopes derived from *Toxoplasma* proteins GRA6, Tgd_057, ROP7 and GRA4 are presented to CD8 T cells (Blanchard et al., 2008; Feliu et al., 2013; Lee et al., 2015). Furthermore, *in vitro* NLRP3 is reported to be crucial for this CD8 T cell mediated immune response (Kongsomboonvech et al., 2020). Mice depleted of CD8 T cells show increased susceptibility in the chronic, but not acute, phase of infection (Denkers et al., 1997). Finally, CD4 and CD8 T cells restrain parasite growth by producing IFNγ, by contact dependent CD40-CD40L interaction mediated autophagy or by perforin-mediated lysis of infected cells (Gazzinelli et al., 1992; Denkers et al., 1997; Reichmann et al., 2000).

### Species Differences in IFNγ-Mediated Parasite Elimination

The primary mechanism of IFNγ-induced cell autonomous immunity against *Toxoplasma* in murine cells is the induction of members of the p47 and p65 family of GTPases (also known as immunity related GTPases, or IRGs, and guanylate binding proteins, or GBPs, respectively). By acting cooperatively, these cause the disruption of the PV, leaving the parasites exposed to the host cell cytoplasm (Taylor et al., 2000; Collazo et al., 2001; Pawlowski et al., 2011; Yamamoto et al., 2012). This, in turn induces autophagy through ubiquitin and autophagy related proteins (ATG) dependent mechanisms (Ling et al., 2006; Choi et al., 2014), as well as the production of reactive oxygen and nitrogen species (Langermans et al., 1992; Arsenijevic et al., 2001) (Figure 1). Detailed mechanisms by which these pathways eliminate *Toxoplasma* have been reviewed elsewhere (Saeij and Frickel, 2017; Evans et al., 2018; Sasai and Yamamoto, 2019). The mechanism of IFNγ-induced cell autonomous immunity varies between murine and other hosts (Gazzinelli et al., 2014; Saeij and Frickel, 2017; Sasai and Yamamoto, 2019). For instance, while in murine cells the IRGs play a dominant role in killing *Toxoplasma*, humans and other animals do not have IFNγ-inducible IRGs (Hunn et al., 2011; Gazzinelli et al., 2014; Müller and Howard, 2016). Furthermore, in murine cells GBPs are recruited and cause the disruption of the PVM, while in human cells GBPs are not recruited to the PVM in most cell types except in mesenchymal stromal cells (MSCs), and are hypothesized to “work from a distance” (Yamamoto et al., 2012; Niedelman et al., 2013; Haldar et al., 2015; Selleck et al., 2015; Johnston et al., 2016; Qin et al., 2017; Saeij and Frickel, 2017; Fisch et al., 2019a). In many IFNγ-stimulated human and murine cell types, ubiquitin and its downstream adaptor proteins p62 and LC3B are recruited to the PVM resulting in parasite growth restriction either by endolysosomal fusion, inhibition of parasite replication or through further GBP-mediated parasite killing (Degrandi et al., 2013; Selleck et al., 2013, 2015; Choi et al., 2014; Haldar et al., 2015; Lee et al., 2015; Clough et al., 2016; Kravets et al., 2016; Foltz et al., 2017; Mukhopadhyay et al., 2020b). Furthermore, in human primary fibroblasts and macrophages, IFNγ-induced cell death can also restrict *Toxoplasma* growth (Niedelman et al., 2013; Fisch et al., 2019a). Additionally, in many human and bovine cell types, IFNγ induces indoleamine 2,3 dioxygenase 1 (IDO1)-mediated L-Tryptophan (L-Trp) breakdown, thus restricting *Toxoplasma* growth, since the parasite is auxotroph for this amino acid (Pfefferkorn, 1984; Pfefferkorn et al., 1986; Dai et al., 1994; Nagineni et al., 1996; Heseler et al., 2008; Schmidt et al., 2009; Spekker et al., 2009; Niedelman et al., 2013; Qin et al., 2017; Bando et al., 2018, 2019). By contrast, this pathway did not play any role in parasite growth restriction in murine cells (Schwartzman et al., 1990; Meisel et al., 2011). Nevertheless, IDO1 was induced in the acute phase of disease in murine models (Divanovic et al., 2012; Ufermann et al., 2019). Furthermore, in mice chronically infected with *Toxoplasma*, inhibition of IDO1 leads to encephalitis (Divanovic et al., 2012). This is possibly due to the fact that L-Trp acts as an essential element for T cell proliferation; indeed regulatory T cells (Tregs) often induce IDO to control excessive T cell proliferation (Puccetti and Grohmann, 2007; Yan et al., 2010). In rat enterocytes, parasite growth is inhibited in an iron-dependent way (Dimier and Bout, 1998). Some of these toxoplasmacidal mechanisms could be especially important in hosts that do not express functional GBP1, GBP2, or GBP5 (Table 1).

## *Toxoplasma* Effectors

*Toxoplasma* strains differ remarkably in virulence and in the immune response they trigger (Sibley and Boothroyd, 1992; Melo et al., 2011; Hunter and Sibley, 2012; Su et al., 2012). *Toxoplasma* differences in attraction of particular cell types might explain variations in their capacity to disseminate, replicate or survive. This, in turn could directly contribute to strain-specific access to immunoprivileged sites such as the central nervous system or the fetus (Saeij et al., 2005). For example, type I strains exhibit a superior extracellular migratory capacity compared to type II or III strains (Barragan and Sibley, 2002). However, type II and, to a lesser extent, type III strains have an increased ability to use DCs as Trojan horses for dissemination compared to type I (Lambert et al., 2009). The difference in virulence between the major strains of *Toxoplasma* is due to multiple polymorphic effectors that act in concert to modulate murine immune responses at different steps, a subject that has been reviewed in-depth elsewhere (Melo et al., 2011; Boothroyd and Hakimi, 2020). The strain variation in specific phenotypes is not only limited to clonal types but is also present within the same haplogroups, possibly due to the presence of polymorphic effectors or differential expression of effector proteins (Khan et al., 2009; Melo et al., 2013; Yang et al., 2013). For instance, while the lab adapted type I RH strain does not activate NFκB due to the lack of a functional version of the polymorphic effector GRA15, another type I strain, GT1, does activate NFκB, as it expresses a functional GRA15 (Khan et al., 2009; Yang et al., 2013). RH also exhibits greater extracellular viability, faster replication and increased resistance against IFNγ compared to GT1, in which GRA15 recruits more p62 and LC3B on the PVM and promotes autophagy by interacting with TRAF6 (Khan et al., 2009; Mukhopadhyay et al., 2020b). As most studies use RH as the prototype for type I strains, one should be careful when extrapolating results to other type I strains (Khan et al., 2009; Yang et al., 2013). Similarly, these intra-clonal group variations are also observed within type II strains; for example the ME49 and Pru strains differ in the induction of CD40 on macrophages due to different GRA15 expression levels likely caused by polymorphisms in its promoter region (Morgado et al., 2014) (see Table 2 for other strain-specific effectors).

**Table 2:** Strain-specific *Toxoplasma* effectors and their effects on the murine immune response.

**Factor**	**Type I**	**Type II**	**Type III**	**Effects on the host**	**References**
ROP18	Active	Active	Not expressed	Phosphorylation of IRGs leading to reduced recruitment to the PVM and parasite resistance to IFNγ-mediated killing. Causes phosphorylation of the ER protein ATF6β, resulting in its proteasomal degradation and thus lowered antigen presentation	Saeij et al., 2006; Taylor et al., 2006; Zhao et al., 2009; Fentress et al., 2010; Khaminets et al., 2010; Steinfeldt et al., 2010; Yamamoto et al., 2011; Niedelman et al., 2012
ROP5	Active	Less active	Active	Binds IRGA6 and inhibits its loading to PVM. Allosteric activation of ROP18, leading to resistance of parasites against IFNγ	Behnke et al., 2011, 2012; Reese et al., 2011; Fleckenstein et al., 2012; Niedelman et al., 2012; Murillo-León et al., 2019
ROP16	Active	Less active	Active	Phosphorylation and sustained activation of STAT3 and STAT6, thus decreasing production of IL12 and induction of arginase. Promotes parasite growth by reducing the PVM loading of GBPs, thus preventing IFNγ-mediated parasite killing	Saeij et al., 2007; Yamamoto et al., 2009; Ong et al., 2010; Butcher et al., 2011; Jensen et al., 2011; Virreira Winter et al., 2011; Fisch et al., 2019b
ROP38	Lowly expressed	Expressed	Highly expressed	Downregulates host genes associated with MAPK signaling and control of apoptosis and proliferation. Induces IL18 secretion	Peixoto et al., 2010; Melo et al., 2013; Xu et al., 2018
GRA6	Active	Less active but has Ld T-cell epitope	Active	Activates the transcription factor NFAT4 via calcium modulating ligand (CAMLG). Type II GRA6 contains the most abundant Ld epitope which results in stronger T-cell responses	Ma et al., 2014
GRA15	Inactive^a^	Active	Lowly expressed	Activates the transcription factor NFκB via TRAF2 and TRAF6. Helps with recruitment of autophagy adaptor proteins p62 and LC3B, as well as IRGs and GBPs, via TRAF6 in IFNγ-stimulated cells, leading to increased parasite susceptibility to IFNγ	Rosowski et al., 2011; Virreira Winter et al., 2011; Sangaré et al., 2019b; Mukhopadhyay et al., 2020b
GRA25	Not determined	Active	Less active	Induces secretion of chemokines CCL2 and CXCL1 by infected macrophages	Shastri et al., 2014
MAF1	Active	Inactive	Active	Mediates host mitochondria association with the PVM. Induces secretion of IL4, IL10, IL13, GCSF, IL6, and IFNγ	Pernas et al., 2014

a *Among type I strains RH has a truncated version of GRA15, while GT1 has a functional GRA15 and can therefore activate NFκB (Rosowski et al., 2011; Yang et al., 2013)*.

It is well-known that type II strains induce a much stronger pro-inflammatory response compared to type I and III strains (Robben et al., 2004; Jensen et al., 2011). Quantitative trait locus (QTL) analysis of host gene expression levels after infection with F1 progenies derived from IIxIII crosses led to the discovery that ROP16 (encoding a secreted kinase) (Saeij et al., 2007) and GRA15 (Rosowski et al., 2011) determine most *Toxoplasma* strain differences in the modulation of the inflammatory response in mice. Through the NFκB pathway, type II GRA15 (GRA15_II_) induces the production of inflammatory cytokines such as IL12 and IL1β, and elicits classically activated macrophages (M1) (Jensen et al., 2011; Gov et al., 2013; Mukhopadhyay et al., 2020a). These M1 macrophages are generally good at killing intracellular pathogens but their secretion of pro-inflammatory cytokines can cause pathology through an excessive activation of NK, Th1, and Th17 cells, and by inducing pro-apoptotic pathways (Murray et al., 2014). On the other hand, type I/III ROP16 (ROP6_I/III_) phosphorylates the transcription factors STAT3/6, driving macrophages toward an alternatively activated (or M2) state that is associated with increased IL10 expression and a Th2-polarized response (Yamamoto et al., 2009; Butcher et al., 2011; Jensen et al., 2013; Chen et al., 2020). These M2 macrophages are less efficient in killing intracellular pathogens (Murray et al., 2014). In addition, ROP16 can also inhibit GRA15-mediated activation of NFκB (Jensen et al., 2013). GRA24 binds to p38 mitogen activated kinase (p38 MAPK), leading to its phosphorylation, nuclear translocation and persistent activation (Braun et al., 2013). GRA24 induces inflammatory cytokine and chemokine secretion from macrophages, resulting in immune activation. All three major clonal populations of *Toxoplasma* express GRA24, however, it is in type II strains where GRA24, together with GRA15 and in the absence of active ROP16, can induce a heightened inflammatory immune response. GRA15 and GRA24-induced IL12 secretion by macrophages is achieved through the activation of the cREL NFκB pathway (Mukhopadhyay et al., 2020a). GRA15 and GRA24 together induce the secretion of IL18 and IL12, resulting in higher IFNγ secretion and lower parasite burden, while infection with parasites lacking these two effectors results in significantly lower IFNγ secretion and concomitantly higher peritoneal parasite burden in comparison to wild-type parasites (Mercer et al., 2020; Mukhopadhyay et al., 2020a). Thus, by inducing the IL12-IL18-IFNγ axis of host immunity, type II strains keep parasite numbers controlled, enabling persistence and further transmission to other hosts. Notably, while type I strains cause mortality primarily due to faster replication and uncontrolled parasite proliferation, type II strains can also cause disease due to their ability to induce an excessive pro-inflammatory response in the host that may lead to ileitis or encephalitis.

## Toxoplasmic Ileitis in Mice

**Figure 2:**
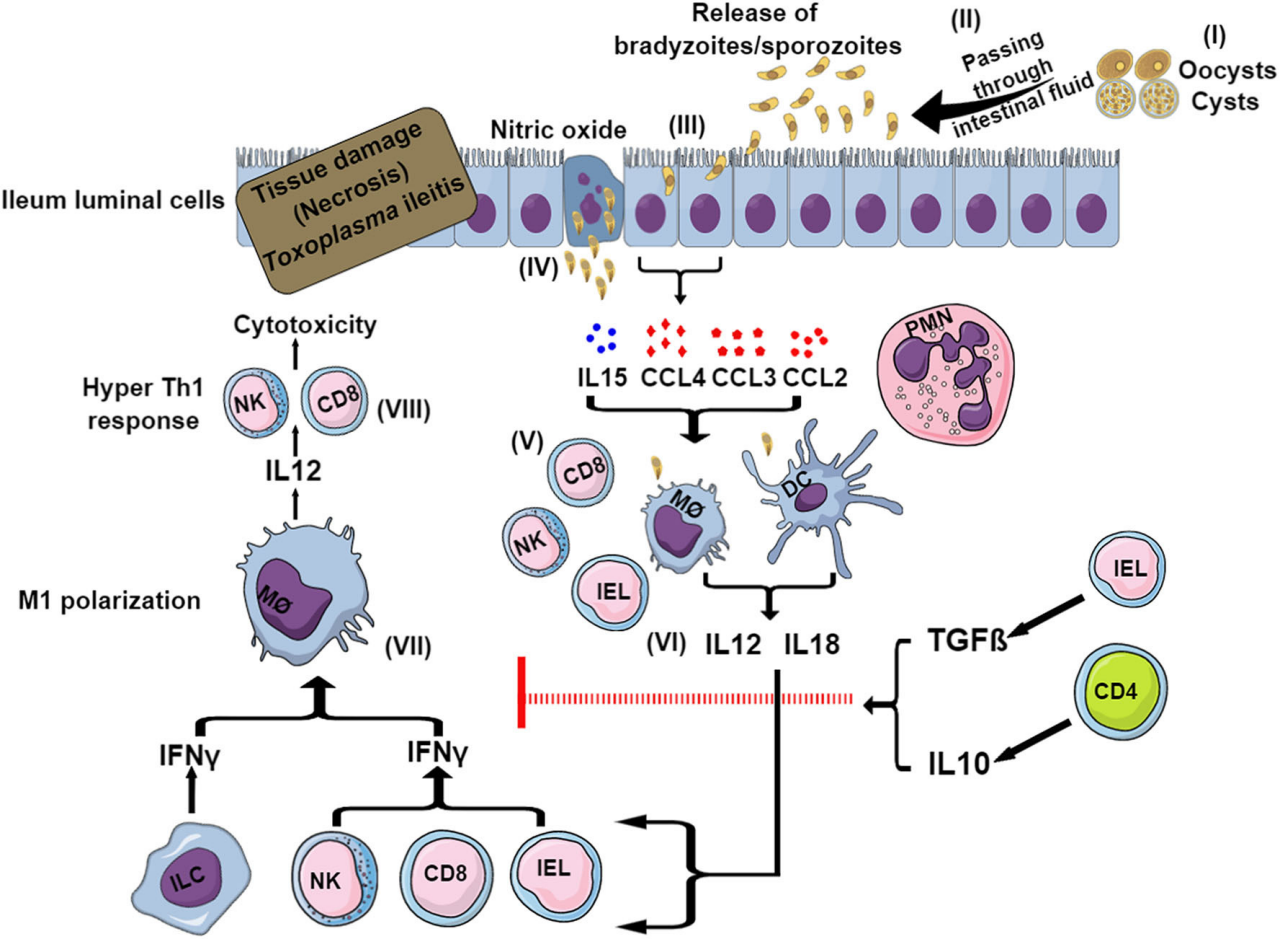
Model for sequential events during *Toxoplasma-*induced ileitis. Following ingestion of oocysts or tissue cysts, digestive enzymes break their wall and sporozoites or bradyzoites, respectively, are released (I). After invading intestinal epithelial cells, bradyzoites and sporozoites convert to tachyzoites (II) (III). Infected enterocytes release cytokines, chemokines and nitric oxides (IV) that attract and recruit neutrophils, macrophages, DCs, CD4, and CD8 T cells, NK cells, IELs, and ILCs (V). DCs and macrophages produce IL12 and IL18 (VI) that in turn stimulates ILCs, IELs, CD8 T cells and NK cells to secrete IFNγ (VII). This IFNγ causes the polarization of macrophages to the pro-inflammatory M1 phenotype. M1 macrophages release more IL12, which exacerbates the Th1-induced immunity (VIII), resulting in necrosis and tissue damage ultimately leading to ileitis. To control this exacerbated immune response, in certain murine strains (BALB/c) IELs and CD4 T cells are able to produce IL10 and TGFβ, which limit the inflammation and thus prevents the development of ileitis.

Within 8 days of oral ingestion of *Toxoplasma* tissue cysts or oocysts, susceptible mouse strains such as C57BL/6 develop severe ileitis resulting in necrosis of mucosal villi and tissue destruction. This is not limited to murine models, as several other animals infected naturally or experimentally with *Toxoplasma* have been reported to develop ileitis (Schreiner and Liesenfeld, 2009). This ileitis is not associated with high parasite burdens but rather with a strong Th1-biased immune response characterized by an overproduction of pro-inflammatory mediators including IFNγ, TNFα, IL1β, IL18, and NO, which is commonly known as “cytokine storm” (Liesenfeld et al., 1996, 1999). The initiation of the immune response starts within the small intestine, where the parasites first invade enterocytes. These cells secrete a variety of chemokines and cytokines, such as CCL2, CCL3, CCL4,and IL15, that attract immune cells including intraepithelial lymphocytes (IELs) (Khan and Kasper, 1996; Buzoni-Gatel et al., 1997; Mennechet et al., 2002) (Figure 2). Intestinal innate lymphoid cells (ILCs) also contribute to the immune response upon oral infection. Mice lacking the transcription factor T-bet have impaired production of IFNγ due to a deficiency of ILC1, failing to control parasite growth despite having NK cells (Klose et al., 2014). Furthermore, while macrophages and DCs mostly produce IL12 and IL18, in turn activating cytotoxic NK cells, CD8 T cells and IELs produce more IFNγ (Egan et al., 2009, 2011) (Figure 2). The effects of IL15 involve DC-mediated production of IL12 and subsequent priming and activation of CD4 and CD8 T cells (Khan et al., 2002; Combe et al., 2006). Different CD4 helper T cell subsets play a dominant role in this Th1-biased immunopathology during ileitis by secreting the inflammatory cytokines IL17 and IL22 (Muñoz et al., 2009; Guiton et al., 2010).

During *Toxoplasma*-induced ileitis, dramatic changes in the commensal gut flora have been observed, bearing a resemblance to inflammatory bowel disease (IBD) (Liesenfeld, 2002). The disturbance of the gut flora has been observed from day 5 post-infection (p.i.) when the epithelial barrier of the intestine shows morphological changes (Heimesaat et al., 2006). High abundance of gram negative bacteria in the intestine and their translocation into subepithelial tissues after oral infection with *Toxoplasma* increased TLR2- and TLR4-mediated sensing and amplified inflammation (Heimesaat et al., 2006, 2007). Thus, this breaching of the intestine by the gut flora acts as a molecular adjuvant that exacerbates the Th1 response regardless of the parasites. The role of the gut flora in *Toxoplasma*-induced ileitis was further demonstrated by two additional findings; firstly, prophylactic or therapeutic administration of antibiotics (ciprofloxacin and metronidazole) against the overgrowing gram negative bacteria improves the immunopathology by reducing the cytokine storm (Heimesaat et al., 2006); secondly, gnotobiotic mice do not develop ileitis following oral infection with *Toxoplasma* (Heimesaat et al., 2006). Furthermore, TLR11 deficient mice display no detectable pathology in the small intestine and other tissues, suggesting that TLR11 is responsible for the immunopathological response during toxoplasmosis (Benson et al., 2009). Additionally, when *Tlr11*^−/−^ mice were infected with increasing doses of *Toxoplasma*, a significantly lower mortality was observed in comparison to wild-type mice (Benson et al., 2009). Nevertheless, how TLR11 contributes to toxoplasmic ileitis immunopathology remains elusive, as intraperitoneal infection of *Tlr11*^−/−^ mice leads to severe pancreatitis, fat necrosis and systemic increase of inflammatory cytokines, which is at least partially related to Caspase1-mediated inflammasome driven IL18 secretion (Yarovinsky et al., 2008). The underlying mechanism of the opposing behaviors in*Tlr11*^−/−^ mice infected orally or intraperitoneally deserves further investigation. Apart from TLR2, TLR4, and TLR11, TLR9 also contributes to the ileitis immunopathology after oral infection with *Toxoplasma* (Minns et al., 2006; Benson et al., 2009). This is not surprising, as both parasite and bacterial DNA can activate the TLR9-signaling pathway. However, contradictory reports indicate that although wild-type and *Tlr9*^−/−^ mice exhibit a comparable degree of ileitis upon oral *Toxoplasma* infection, the latter develops a higher parasite burden and higher IFNγ and NO levels (Bereswill et al., 2014). Although, the reasons for this discrepancy are not known, it is possible that an altered gut flora is present in mice bred at different colonies or acquired from different vendors, as previously observed by others (Ericsson et al., 2015; Alegre, 2019).

### Control of Toxoplasmic Ileitis Related Immunopathology

To curb the hyper-inflammatory milieu generated by the gut flora and TLR-induced signaling, CD4 T cells play an important role by producing regulatory IL10, which causes a downregulation of IFNγ-induced responses (Figure 2). As a matter of fact, CD4 T cell and IL10 knockout mice succumb rapidly to oral cyst infection (Gazzinelli et al., 1996). Furthermore, IELs produce transforming growth factor (TGF)β, which downregulates the IFNγ response and inhibits chemokine production from enterocytes, thereby limiting the recruitment of more immune cells and reducing inflammation (Buzoni-Gatel et al., 2001; Mennechet et al., 2004) (Figure 2). IL27, another anti-inflammatory cytokine, also helps in reducing the inflammation. This was evident in WSX-1 (the IL27 receptor) deficient mice which displayed more severe immunopathology after *Toxoplasma* oral infection despite having a similar parasite burden compared to wild-type mice (Villarino et al., 2003). IL27 controls immunopathology by promoting IL10 production, by inhibiting CD4 T cell proliferation, by decreasing IL2 production and possibly also by increasing the inhibitory ligand programmed cell death ligand 1 (PD-L1) on CD4 T cells (Dupont et al., 2012).

### *Toxoplasma*-Induced Ileitis Is Dependent on the Mouse Genetic Background

The aforementioned gut immunopathology has been observed in susceptible C57BL/6 mice, while BALB/c mice are in general resistant (Johnson, 1984; McLeod et al., 1989a; Liesenfeld et al., 1996; Subauste, 2012). This could be explained by the fact that T cells from C57BL/6 mice preferentially secrete Th1 cytokines with high IFNγ and low IL4 production, whereas those from BALB/c mice favor Th2 cytokine production with low IFNγ and high IL4 secretion (Hsieh et al., 1995). Moreover, BALB/c mice produce more anti-inflammatory IL10 to dampen the Th1 immunity (Suzuki et al., 2000). On the other hand, the fatal outcome upon oral infection in C57BL/6 mice is mainly caused by an excessive intestinal tissue damage produced by an hyper-inflammatory immune reaction and lower IL10 production that leads to a leakage of gut flora and a cytokine storm (Suzuki et al., 2000). Furthermore, macrophages from BALB/c mice produce less IL12 and TNFα than those from C57BL/6 mice when stimulated with *Toxoplasma* antigen or live parasites, as well as other microbial ligands (Schade and Fischer, 2001; Watanabe et al., 2004). BALB/c mice also produce significantly less IFNγ than C57BL/6 mice upon *Toxoplasma* infection (Shirahata et al., 1986). However, whether this also applies to *Toxoplasma*-induced ileitis is currently unknown and warrants further investigation.

## Toxoplasmic Encephalitis

During the chronic phase, *Toxoplasma* cysts are controlled, but not eliminated, remaining largely quiescent for the life of the host. In immunocompromised patients, such as those with advanced AIDS, transplanted or under chemotherapy, cysts can reactivate and cause toxoplasmic encephalitis (TE). This progressive inflammatory disease is characterized by cyst rupture, tachyzoite conversion and parasite replication within the CNS, which often results in a fatal outcome in the absence of treatment (Blanchard et al., 2015; Schlüter and Barragan, 2019).

### Clinical Signs

Clinical manifestations include headaches, fever, hemiparesis, ataxia, and seizures, among others (Navia et al., 1986; Porter and Sande, 1992; Marra, 2018). Moreover, one of the most intriguing effects of *Toxoplasma* chronic infection is the behavioral changes produced in various hosts (Berdoy et al., 2000; Webster, 2007; Webster et al., 2013; Boillat et al., 2020). This has been mainly investigated in mice, in which a variety of effects have been described, ranging from impaired movements, deficits in spatial learning and memory, higher activity and explorative behavior, reduced anxiety, and, most notably, reduced aversion to feline odors (Vyas, 2015; Tedford and McConkey, 2017; Schlüter and Barragan, 2019). This later phenomenon is commonly referred to as “fatal attraction” and in nature it is thought to enhance the likelihood of *Toxoplasma* transmission to its definitive feline host (Berdoy et al., 2000; Giacomini et al., 2007). Although it has been long assumed that this attraction was specific to felids, a recent report showed that *Toxoplasma*-infected mice lose their innate fear to several other animal odors and unknown objects as well (Boillat et al., 2020). Notwithstanding, the decreased aversion seems to be caused by complex mechanisms altering the perception of the host. Local neural tissue destruction caused directly through parasite multiplication or indirectly by an excessively inflammatory immune response may produce adverse effects. This is usually considered marginal, given the low density of cysts and the fact that they are randomly scattered in different areas of the CNS (Vyas and Sapolsky, 2010; Berenreiterová et al., 2011; Tedford and McConkey, 2017). The absence of a specific tropism for brain regions points toward a behavioral manipulation mediated by neuroinflammation rather than by direct interference of the parasite itself with specific areas of the brain (Martynowicz et al., 2019). In support of this, a positive correlation has been observed between the severity of behavioral alterations and the level of inflammation, which in turn is reflected by cyst loads (Boillat et al., 2020).

The cells and effectors involved in an adequate control of *Toxoplasma* and tissue damage in the brain are discussed in detail below. Nevertheless, the production of pro-inflammatory cytokines may alter the levels of several neuromodulators, including dopamine, glutamate and serotonin, thereby affecting the behavior (Webster and McConkey, 2010; Berenreiterová et al., 2011; David et al., 2016; Babaie et al., 2017). Other studies have also described that the parasite itself can influence neurotransmitter levels such as dopamine, likely contributing to the hyperactivity and enhanced novelty seeking in infected rodents (Skallová et al., 2006; Prandovszky et al., 2011). It is important to remark that data obtained in murine models do not necessarily apply to humans or other animals, since the immune response against *Toxoplasma* greatly differs as outlined earlier. Although it is out of the scope of this review, a considerable number of epidemiological studies have linked *Toxoplasma* infection with several mental illnesses such as schizophrenia, Parkinson's and bipolar disorders (Fabiani et al., 2015). However, there is no consensus as to whether *Toxoplasma* is a risk factor to mental illnesses or mental illnesses are risk factors to *Toxoplasma* infection. Moreover, these mental illnesses in humans are surely influenced by many other factors, which is also reflected by the lack of correlation between the incidence of mental illnesses and *Toxoplasma* prevalence in different regions of the world.

### Blood-Brain Barrier, Cyst Location, and Cell Tropism

How *Toxoplasma* reaches the brain and invades neural tissues in the infected host is not completely understood. It is assumed that during the acute phase of infection tachyzoites are transported in the bloodstream/lymph and disseminate through the body either directly as extracellular parasites or by exploiting infected leukocytes as Trojan horses. In this process, the parasite *Tg*WIP rhoptry protein enhances motility and transmigration abilities of infected DCs (Sangaré et al., 2019a). This effector is essential for the parasite to reach the brain, as mice infected with knockout parasites do not have cysts in the brain, at least after infection with the type II ME49 Δ*Tgwip* strain (Sangaré et al., 2019a). Whether this applies to other strains or hosts, and the possible role of other virulence factors deserves further research. Regardless, once the parasite reaches the brain vasculature, it is believed that it can cross the blood-brain barrier (BBB) either as intracellular parasites using transmigrating infected cells as deliverers or directly as extracellular tachyzoites by transcytosis or paracytosis (Blanchard et al., 2015; Konradt et al., 2016; Wohlfert et al., 2017). A growing body of evidence suggests that rather than using infected leukocytes as true Trojan horses to cross the BBB, *Toxoplasma* uses them to reach the cerebral vasculature, subsequently egressing to contact endothelial cells and finally extracellular parasites directly cross the BBB following paracellular mechanisms (Drewry and Sibley, 2019). Indeed, it seems that parasites need to invade, replicate and lyse cerebral endothelial cells in order to reach the brain parenchyma (Konradt et al., 2016). In addition, cells infected with the non-replicating CPS strain were not observed in the brain after intravenous injection, supporting the notion that although the trojan horse mechanism is important for dissemination, it may not contribute to the crossing of the BBB. The ROP17 kinase promotes an integrin-independent interstitial migration of infected monocytes while inhibiting the integrin-dependent cross of biological barriers such as the BBB (Drewry et al., 2019). Hence, it is likely that, besides *Tg*WIP, ROP17 is also important in type II and III strains (which have reduced extracellular dissemination abilities and rely mostly on the Trojan host mechanism) to reach the brain or other distant organs (Lambert et al., 2009). In support of this possibility, a recent study showed that the formation of tissue cysts in the brain of infected mice is almost completely abrogated in the type II Pru Δ*rop17* strain, which also had a decreased virulence (Fox et al., 2016). Taken together, these results suggest that the integrin-dependent crossing of the BBB (naturally inhibited by ROP17) is not the main mechanism used for type II strains, as otherwise it would be expected that in the absence of ROP17 the parasite had an enhanced transmigration through the BBB. However, it is also possible that the parasites are not even able to reach the brain because of the lack of ROP17-induced promotion of monocyte migration, or that other factors or mechanisms are also involved. For instance, the early egress phenotype observed *in vitro* for Pru Δ*rop17* parasites may preclude bradyzoite differentiation *in vivo* (Fox et al., 2016). Be that as it may, since these mechanisms are likely influenced by the host and strain involved in the infection (Barragan and Sibley, 2002; Lambert et al., 2009), further studies are warranted to investigate how *Toxoplasma* crosses the BBB.

Even under the same experimental conditions, the number and location of cysts largely varies among individuals (Berenreiterová et al., 2011; Boillat et al., 2020). However, a number of facts are consistently reported. Firstly, cyst numbers decline with time, likely due to reactivation and destruction by the immune system (Melzer et al., 2010; Watts et al., 2015), although the frequent observation of clusters of cysts of varying sizes also indicate that new or second-generation cysts can be formed during chronic infections (Melzer et al., 2010; Berenreiterová et al., 2011). Secondly, the distribution of cysts is bilateral, widespread and non-homogeneous, indicating an absence of targeted areas. Although there is no clear consensus on which regions are usually more affected, generally the gray matter and the cerebral cortex, especially the frontal cortex, harbors the great majority of cysts, while consistently low numbers are seen in the cerebellum (Melzer et al., 2010; Berenreiterová et al., 2011; Pittman et al., 2014; Boillat et al., 2020). Since the olfactory bulbs are contained in the frontal cortex region, the higher presence of the parasite in this area could explain the loss of feline odor aversion in infected mice (Berdoy et al., 1995; Torres et al., 2018). However, it appears that the accumulation of cysts in this region is not sufficient to account for this effect, but rather it seems to be a consequence of complex mechanisms altering the perception of the host (Boillat et al., 2020). Finally, cysts are found almost exclusively in neurons, and these are located in the neuronal processes rather than in the cell body (Ferguson and Hutchison, 1987a,b; Melzer et al., 2010; Cabral et al., 2016). Numerous *in vitro* studies using mouse and human brain cells have shown that, apart from neurons, the parasite is able to infect and encyst in astrocytes, microglia and Purkinje cells (Jones et al., 1986; Fischer et al., 1997; Halonen et al., 1998; Carruthers and Suzuki, 2007). Despite this, studies performed in chronically infected patients have described that, although it is possible to observe cysts in different brain cells, the great majority are found within neurons (Powell et al., 1978; Bertoli et al., 1995). Likewise, practically only neurons harbored cysts in chronically infected mice (Melzer et al., 2010; Cabral et al., 2016; Schlüter and Barragan, 2019). Why *Toxoplasma*, which is able to virtually invade all nucleated cells, has this predilection for neurons remains unclear. It is possible that the limited capacity of neurons for antigen presentation and responsiveness to IFNγ stimulation, together with their extensive size compared to other cells, provides the parasite with a safe niche to avoid the immune response and form large cysts, as opposed to astrocytes, which efficiently destroy the parasite (Schlüter et al., 2001; Blanchard et al., 2015; Cabral et al., 2016; Wohlfert et al., 2017).

### Immune Response in the Brain During Chronic Infection

During the chronic phase of infection, a continuous and effective immune response is required to keep the parasite under control and prevent the reactivation of cysts (Wohlfert et al., 2017). Hence, under immunocompromised circumstances the periodic cyst rupture and parasite conversion back to tachyzoites are uncontrolled, leading to massive tissue destruction and inflammation of the brain (Carruthers and Suzuki, 2007). This life-threatening TE can also occur in immunocompetent patients in some instances, such as in congenitally infected individuals or infections produced by non-archetypal strains, where an exacerbated immune response produces excessive inflammation leading to tissue damage and neurological signs (Carme et al., 2009; Hamilton et al., 2019).

Mouse models, as well as observations made in AIDS patients, show that the type of immune response required to prevent reactivation in the brain and to control acute infections are similar. Indeed, IFNγ and other pro-inflammatory cytokines are needed to control parasite burden; however without regulatory and anti-inflammatory cytokines the highly activated immune cells filtering into the brain could produce encephalitis regardless of parasite burden (Landrith et al., 2015). CD8 and, to a lesser extent, CD4 T cells play a critical role in controlling infection in the brain during chronic toxoplasmosis (Gazzinelli et al., 1992; Luft and Remington, 1992). CD8 T cells secrete cytokines involved in parasiticidal effects, in particular IFNγ and TNFα (Fischer et al., 2000; Schlüter and Barragan, 2019), and can directly control cyst burdens in the CNS via perforin-mediated cell lysis, although this effect seems to be limited and independent of IFNγ (Denkers et al., 1997; Wang et al., 2004; Suzuki et al., 2010, 2011; Landrith et al., 2015). In the course of TE with ongoing inflammation, the permeability of the BBB is likely high and accompanied by a continuous influx of immune cells from the periphery (Blanchard et al., 2015). These inflammatory infiltrates are mainly composed of CD4 and CD8 T cells, as well as macrophages, CD11c+ DCs, and Ly6C^high^ monocytes (Schlüter and Barragan, 2019). Immune cells may cross the BBB through a process involving slowing, adhesion and extravasation (diapedesis) into the perivascular space. This is achieved by their interaction with several molecules expressed on endothelial cells of the cerebral vasculature, including adhesins, integrins, selectins, or metalloproteinases (Wilson et al., 2010; Clark et al., 2011; Suzuki et al., 2011; Landrith et al., 2015). Among them, the vascular cell adhesion molecule 1 (VCAM-1) plays a pivotal role in the recruitment of T-cells to the CNS by interacting with its main ligand VLA-4, an α4β1 integrin that is expressed on activated T-cells (Carrithers et al., 2000; Wang et al., 2007). Moreover, increased expression of VCAM-1 in cerebral vessels is partially dependent on IFNγ (and possibly TNFα and IL1β) levels, acting as a pro-inflammatory feedback mechanism (Wang et al., 2007; Suzuki et al., 2011). Leukocytes can also migrate to the interstitium using integrin-independent mechanisms, such as blebbing, deformation-based movement and membrane flow (Fackler and Grosse, 2008; Renkawitz and Sixt, 2010; Paluch et al., 2016). Different studies have shown that this integrin-independent migration occurs for DCs, neutrophils and T-cells (Woolf et al., 2007; Lämmermann et al., 2008, 2013). Apart from the aforementioned role in the recruitment of T-cells into the brain through the increased expression of VCAM-1 in endothelial cells, IFN-γ can activate other non-T cells such as microglia, astrocytes and macrophages, in turn developing different mechanisms to control the proliferation of the parasite (Suzuki et al., 2005, 2011). Moreover, astrocytes, microglia and neurons residing in the brain contribute to the control of TE by the production of cytokines, chemokines and by expressing immunoregulatory cell surface molecules (Blanchard et al., 2015).

Several *in vitro* and *in vivo* studies have shown that IFNγ-activated astrocytes control TE in a STAT1-dependent manner and are able to produce different cytokines and chemokines such as IL1β, IL-6, granulocyte-macrophage colony-stimulating factor (GM-CSF), CXCL10 or CCL2 (Fischer et al., 1997; Strack et al., 2002; Hidano et al., 2016; Wohlfert et al., 2017; Schlüter and Barragan, 2019). Astrocytes have also a role in limiting the lesions size and necrosis by encircling inflammatory infiltrates around cysts (Wilson and Hunter, 2004; Drögemüller et al., 2008; Melzer et al., 2010). Other *in vitro* reports have observed that different mediators act on astrocytes depending on the host. For example, in human astrocytes and glioblastoma cells, tachyzoites are killed through nitric oxide (NO) and its growth is inhibited by the upregulation of IDO through the synergistic action of IFNγ and TNFα (Däubener et al., 1996; Suzuki et al., 2011; Bando et al., 2019). In mice, on the other hand, is seems that the *Toxoplasma* inhibitory effects of astrocytes are mediated by IRGM3 (IGTP) and IRGA6 (IIGP1), which disrupt the PV (Halonen et al., 1998, 2001; Martens et al., 2005; Melzer et al., 2008). Brain resident macrophages, also known as microglia, are also important in the control of TE, as these are the only brain cells that express MHC-II, thus acting as professional APCs that tune T-cell protective responses (Blanchard et al., 2015). Similar to astrocytes, both NO-dependent and independent mechanisms appear to be involved in the inhibitory effects of microglia on TE, at least in mice (Chao et al., 1993; Deckert-Schlüter et al., 1999; Schlüter et al., 1999; Rozenfeld et al., 2005). IFNγ-activated microglia also produce TNFα that exerts an important effect on the resistance against parasite reactivation during chronic toxoplasmosis (Blanchard et al., 2015). Moreover, a distinct population of alternatively activated brain macrophages/microglia are able to destroy *Toxoplasma* cysts by secreting the acidic mammalian chitinase (AMCase), an enzyme produced in response to the structural chitin present in the cyst wall (Nance et al., 2012). The chitin-mediated attack by these M2 macrophages releases parasites from latent cysts, providing a constant antigenic stimulation for the immune response and likely explaining the continuous recruitment of T cells into the brain during chronic toxoplasmosis. Microglia are also critical during TE for their immunoregulatory properties mediated by GM-CSF and TGFβ to avoid excessive inflammation and tissue damage (Hunter et al., 1992; Chao et al., 1993, 1994; Fischer et al., 1993). Finally, infiltrating DCs and NK cells are also important to control TE (Landrith et al., 2015). The former are the main producers of IL12, which is crucial for the maintenance of IFNγ production by T-cells, and may also act as APCs to further activate T-cells (Fischer et al., 2000; John et al., 2011; Blanchard et al., 2015). The role of NK cells in *Toxoplasma* chronic infection and TE is not well-understood, although it is possible that they are recruited to the brain and represent an important source of IFNγ (Suzuki, 2002; Pittman et al., 2014).

To prevent immunopathology while controlling parasite reactivation, a delicate balance between a protective pro-inflammatory and a dampening regulatory response is needed. In this sense, immunosuppressive and regulatory cytokines are important for the downregulation of inflammatory reactions during the chronic stage of *Toxoplasma* infection. For instance, recruited monocytes secrete IL10 to prevent an excessive immunopathological response in the inflamed brain during *Toxoplasma* chronic stage in mice (Deckert-Schlüter et al., 1997; Biswas et al., 2015). Similarly, astrocytes have been shown to produce IL27, which is critical to suppress the development of Th17 cells and inhibit the toxic effects of IL17 occurring in TE (Stumhofer et al., 2006; Schlüter and Barragan, 2019). TGFβ signaling activation in astrocytes and microglia is also important to prevent neuroinflammation during TE (Cekanaviciute et al., 2014). This regulatory cytokine seems to be secreted by infected neurons (Schlüter et al., 2001; Händel et al., 2012). Other studies have described the role of IL33 and Lipoxin A4 (LXA4) in the downregulation of IFNγ and IL12 production, respectively, during *Toxoplasma* chronic infection (Aliberti et al., 2002; Machado and Aliberti, 2006; Jones et al., 2010).

### Influence of the Host on Toxoplasmic Encephalitis

As mentioned above, the cytokine-induced mechanisms taking place during TE differ between animal species. Most studies have been conducted in mice and humans, and even within mice, the functional relevance of anti-parasitic effector molecules depends on the mouse strain (Schlüter and Barragan, 2019). For example, while NO is essential for the control of intracerebral *Toxoplasma* in susceptible C57BL/6 mice, deletion of iNOS in the resistant BALB/c mice does not exacerbate TE (Schlüter et al., 1999). Moreover, the resistance to development of TE in mice is influenced by the genetic background of the strain and was mapped almost 30 years ago to the Ld region of the MHC class I H-2 gene (Suzuki et al., 1991, 1994; Brown et al., 1995). Mice with b (e.g., C57BL/6) or k (e.g., CBA/Ca) alleles at the H-2 region develop severe encephalitis during the chronic stage of *Toxoplasma* infection. By contrast, those mice strains with H-2^a^ (e.g., A/J) or H-2^d^ (e.g., BALB/c) haplotypes are resistant to the infection, harbor fewer cysts in the brain and rarely show signs or excessive inflammation (Suzuki et al., 1991). Hence, mice with the H-2^d^ haplotype would be the most appropriate models to study human chronic toxoplasmosis in immunocompetent patients. The molecular basis underlying this mouse strain difference is that the immunodominant decamer epitope (HF10) located at the C-terminus of GRA6 elicits a strong and protective CD8 immune response through its presentation exclusively by the H-2 L^d^ (Blanchard et al., 2008; Feliu et al., 2013). Besides being very immunogenic, this region of GRA6 presents a high polymorphism between strains; in fact, peptides derived from this region have been successfully used for serotyping techniques (e.g., Kong et al., 2003; Arranz-Solís et al., 2019). Only the type II specific HF10 sequence (HPGSVNEFDF) binds to H-2^d^ in CD8 T-cells, providing protection by controlling parasite burden. This does not occur in the GRA6 type I/III version of the epitope (HY10, HP*ER*VNVFD*Y*) or with the H-2b or k alleles in susceptible mice. Hence, the recognition of the GRA6 HF10 peptide by CD8 T-cells may account for the differences in virulence among mouse strains during chronic toxoplasmosis, and might also explain why subdominant responses, such as CD4 T-cells, could not be sufficient to control the parasite. In humans, some studies pointed out that the MHC II Human Leukocyte Antigen (HLA)-DQ3 and DQ1 could be genetic markers of susceptibility and resistance, respectively, to cerebral toxoplasmosis in AIDS and congenitally infected patients (Suzuki et al., 1996; Mack et al., 1999). Therefore, both in mice and humans the regulation of the immune response by MHC genes appears to be important for the fate of the host during chronic toxoplasmosis.

### Influence of the Parasite Strain on Toxoplasmic Encephalitis

The parasite strain causing the infection plays a major role in the final outcome of chronic toxoplasmosis. The difference in virulence and immune response elicited during the acute phase strongly influences the fate of the host during the chronic infection. Although few studies have directly investigated how specific virulence factors affect *Toxoplasma* infection in the brain, ROP proteins seem to exert a strong effect on the outcome of infection. In a recent study, the role of several ROPs was investigated by infecting mice with individual knockouts in the Pru type II strain and comparing virulence and cyst formation (Fox et al., 2016). ROP16 deletion increased the parasite burden in the brain, while ROP5, ROP17, and ROP18 deletion produced an almost complete ablation of cyst formation. However, one has to be careful with the interpretation of these results, as reduced cyst numbers could be due to fewer parasites reaching the brain, because of increased susceptibility to immune clearance, or because of reduced dissemination to the brain. For example, the ~100-fold decrease in virulence of Δ*rop5* and Δ*rop18* type II strains likely led to fewer parasites reaching the brain (Fox et al., 2016). Similarly, as ROP17 plays an important role in parasite dissemination by increasing infected monocyte migratory capacities (Fox et al., 2016; Drewry et al., 2019) and in the export of GRA effectors beyond the PVM (Panas et al., 2019), it is likely that the defect in cyst formation of Δ*rop*17 parasites was due to decreased dissemination and virulence. In another study, compared to type II Pru-infected mice, type III CEP-infected mice showed a larger number of macrophages/microglia and infiltrating T cells, as well as increased levels of pro-inflammatory cytokines in the brain, while the number of M2 macrophages and Treg cells was lower (Tuladhar et al., 2019). This is in clear contrast to other reports analyzing the acute infection both *in vitro* and *in vivo*, where macrophages infected with type III parasites show M2-polarized phenotypes (Jensen et al., 2011, 2013; Chen et al., 2020). This suggests that the immune response against *Toxoplasma* evolves over time, as previously reported in type I strains (Mordue et al., 2001). Nonetheless, caution is needed when comparing these studies, as Tuladhar et al. (2019) did not directly analyze the phenotype of infected macrophages but rather the entire population.

In addition, macrophages have been shown to play a dual role in initiating and subsequently resolving inflammation with the coexistence of M1 and M2 activation programs in response to *Toxoplasma* infection (Patil et al., 2014). Early after infection, type III-infected mice have more M2 macrophages compared to type II-infected mice, which induce a stronger acute pro-inflammatory response. In CEP-infected mice, on the other hand, the initial response switches to a more pro-inflammatory profile after 21 days p.i., when the chronic infection is starting to establish (Tuladhar et al., 2019). ROP16 was the factor responsible for this change in the immune response over time, as the CEP Δ*rop*16 strain showed a typical type II immune response profile in the brain, with fewer T-cell and macrophage infiltrates and an increased M2 phenotype. Furthermore, CEP Δ*rop*16-infected animals showed a mixed acute immune response with increased pro-inflammatory macrophages but also M2 macrophages and Tregs, hence its rapid clearance and lowered cyst burden compared to the wild-type (Tuladhar et al., 2019). Since type III strains do not express ROP18, they are very sensitive to mouse IRGs (Fentress et al., 2010; Niedelman et al., 2012), which makes ROP16 crucial in these strains to dampen the initial immune response early after infection and thus avoid rapid clearance. In contrast to ROP16_I/III_, the inactive version of ROP16 expressed by type II strains (ROP16_II_) does not seem to be important for the chronic stage of infection in these strains (Fox et al., 2016).

In addition to ROPs, GRA proteins have also been described to play an important role in the chronic phase of infection, some of them in a strain-specific manner. For example, Bando et al., showed that type II GRA15 suppresses IFNγ-mediated anti-*Toxoplasma* responses in several human liver and brain cell lines (including neurons, glioblastoma, and neuroblastoma cell lines) when co-cultured with monocytes. Namely, GRA15 induces the expression of IL1β by monocytes that in turn promotes iNOS expression in the brain cell lines, which inhibits IDO1-mediated tryptophan degradation, thus facilitating parasite proliferation (Bando et al., 2019). Two other factors involved in parasite dissemination in a strain-specific manner are GRA6 and GRA25 via their effect on CCL2 induction (Ma et al., 2014; Shastri et al., 2014). Only the C-terminus GRA6 epitope from type I and III strains elicit NFAT4 activation leading to CCL2 secretion (Ma et al., 2014). However, as mentioned above, this polymorphic epitope is also crucial in the induction of CD8 T cell responses, but in this case the type II version is the one exerting this effect (Blanchard et al., 2008; Feliu et al., 2013). Therefore, it seems that type II strains have high CD8 T cell activation but a reduced NFAT4-dependent attraction of other immune cells. In place of GRA6, type II strains might elicit a production of chemokines and attraction of monocytes through GRA25 (Shastri et al., 2014). Since GRA25 is equally expressed by tachyzoites and bradyzoites, it is possible that it plays an important role in the control of the parasite in the brain during the chronic phase of infection.

In summary, an effective control of TE depends on IFNγ and CD8 T-cells, while a correct immunoregulation is critical to control excessive inflammatory response in the brain. Of note, since human studies cannot be performed *in vivo*, most research on the mechanisms of intracerebral *Toxoplasma* control and immune response have been conducted *in vitro* or in mice. Thus, caution is needed when extrapolating results obtained with these models, as there are significantly different genetic backgrounds and immune mechanisms involved. Moreover, given the strong influence of both the host and the parasite strain on the outcome of chronic toxoplasmosis, it is possible that specific combinations can uniquely affect the course and pathology of the disease. Nevertheless, a further understanding of the association between strain, immune response and clinical manifestation is needed, especially in severe TE or ocular toxoplasmosis. This, together with an accurate diagnosis would in turn allow an individualized monitorization and improved treatments in risk groups.

## Concluding Remarks and Future Directions

Toxoplasmosis is a disease that varies enormously in its clinical form, ranging from asymptomatic cases to lethal consequences. In addition to the importance of *Toxoplasma* infections in livestock that causes important economic losses, it also poses a risk to humans by direct exposure to contaminated animal products. The variable outcome of infection is strongly dependent on the *Toxoplasma* strain producing the infection, the host and its immune response. In the last decades advancements have been made to understand the mechanisms underlying these differences. Although in this review we focused on the current knowledge of host and parasite factors affecting the outcome of the disease and the control of the parasite, most studies have been performed in murine models and using laboratory adapted strains that do not reflect natural conditions of infection. In addition, most effectors that are important in mice for the control of *Toxoplasma* play no role in other animal species, including humans; thus, the results obtained in these models have to be carefully considered and extrapolations are not always possible.

There are still many questions to be answered: for instance, why is there an intrinsic variability between individuals from the same species? Why are some *Toxoplasma* strains extremely virulent in some hosts and totally controlled in others? What is the most critical component of the immune response that makes some hosts resistant to *Toxoplasma* infection? Arguably, many other factors besides the parasite strain and the host also play an important role and account for these differences, such as the infective dose, the immunological status or genetic individual differences. Moreover, although we have focused on toxoplasmic ileitis and encephalitis, *Toxoplasma* also causes other important diseases in humans such as ocular toxoplasmosis, congenital infection or abortions in pregnant women. Each one of the clinical forms deserve individual investigation to uncover the most relevant host and parasite factors affecting the outcome of infection. Finally, experimental models beyond the murine ones would greatly improve our understanding of the host-parasite relation in toxoplasmosis and, more importantly, would help to elucidate the mechanisms of resistance in humans, with potential applications in terms of new vaccine or drug targets.

## Author Contributions

DM, DA-S, and JPJS have contributed to the conceptualization, writing, and editing of this manuscript. All authors contributed to the article and approved the submitted version.

## Conflict of Interest

The authors declare that the research was conducted in the absence of any commercial or financial relationships that could be construed as a potential conflict of interest.
